# The Potential Virulence Factors of* Providencia stuartii*: Motility, Adherence, and Invasion

**DOI:** 10.1155/2018/3589135

**Published:** 2018-02-21

**Authors:** Naziia Kurmasheva, Vyacheslav Vorobiev, Margarita Sharipova, Tatyana Efremova, Ayslu Mardanova

**Affiliations:** ^1^Institute of Fundamental Medicine and Biology, Kazan Federal University, Kazan, Russia; ^2^Interdisciplinary Center for Analytical Microscopy, Kazan Federal University, Kazan, Russia; ^3^Institute of Cytology RAS, Saint Petersburg, Russia

## Abstract

*Providencia stuartii* is the most common* Providencia* species capable of causing human infections. Currently* P. stuartii* is involved in high incidence of urinary tract infections in catheterized patients. The ability of bacteria to swarm on semisolid (viscous) surfaces and adhere to and invade host cells determines the specificity of the disease pathogenesis and its therapy. In the present study we demonstrated morphological changes of* P. stuartii* NK cells during migration on the viscous medium and discussed adhesive and invasive properties utilizing the HeLa-M cell line as a host model. To visualize the interaction of* P. stuartii* NK bacterial cells with eukaryotic cells* in vitro* scanning electron and confocal microscopy were performed. We found that bacteria* P. stuartii* NK are able to adhere to and invade HeLa-M epithelial cells and these properties depend on the age of bacterial culture. Also, to invade the host cells the infectious dose of the bacteria is essential. The microphotographs indicate that after incubation of bacterial* P. stuartii* NK cells together with epithelial cells the bacterial cells both were adhered onto and invaded into the host cells.

## 1. Introduction

Gram-negative bacteria* Providencia stuartii* are ubiquitous in the environment.* P. stuartii* are also known to cause nosocomial infections. Recent studies have shown that the incidence of infections associated with* P. stuartii* among hospitalized patients is approximately 4 cases per 100,000, indicating an overall low level of virulence in these microorganisms [1]. However, in the case of urinary tract infections, this pathogen causes catheter-associated infections in 9% of the cases [2]. Especially dangerous are nosocomial outbreaks caused by multidrug resistant (MDR) strains of* P. stuartii*. In such cases, mortality may reach 30% [3]. In addition to multiple drug resistance, most clinical isolates of* P. stuartii* contain plasmids encoding extended-spectrum beta-lactamases [4].

Many studies have established that bacteria* P. stuartii* are able to migrate from the urinary tract to other organs, causing endocarditis [5], pericarditis [6], peritonitis [7], and meningitis [8]. The organism's ability to spread through organ systems in hospital settings contributes to the growing concern of health professionals and clinical microbiologists [1].

Bacterial pathogens use a number of mechanisms to infect their hosts: adhesion, colonization of tissues, and in some cases induction of their uptake by the cell of the macroorganism. The induced uptake is called invasion. Pathogens use intracellular multiplication to spread in other tissues or persist, since the ability to invade cells helps bacteria to evade host defenses [9].

Bacteria have historically been divided into two distinct groups: extracellular bacteria, which exist as free-living organisms in their environmental niches, and intracellular bacteria, which infect and replicate inside host cells. Facultative intracellular bacteria, including* Salmonella* spp.,* Francisella* spp.,* Legionella pneumophila*,* Listeria monocytogenes*,* Yersinia* spp., and many others, can replicate in either niche [10]. Currently, a growing number of bacteria that were so-far considered extracellular have been shown to invade host cells, probably using intracellular compartments for persistence in target tissues [9].

Johnson et al. (1987) showed that strains of* P. stuartii* differ in adhesive properties with respect to mice uroepithelial cells [11]. Previously, it was also shown that the invasive properties of* Providencia* differ in different species and strains and depend on the type of eukaryotic cells. It has been established that the invasive properties of* Providencia alcalifaciens* depend on multiplicity of infection (MOI) [12]. In addition, the ability to adhere onto uroepithelial cells may play a role in the persistence of* P. stuartii* in the catheterized urinary tract [13]. However, until now, there have not been enough studies that would classify this pathogen as intracellular.

Data presented in this work demonstrate that a clinical isolate of* P. stuartii* is able to adhere to and invade HeLa-M epithelial cell line. The study of these factors is important for understanding the molecular mechanisms of pathogenesis and the control of infections caused by these bacteria. In addition,* P. stuartii* bacteria can be used as a model of the interactions between opportunistic bacteria and host cells, which are still poorly understood.

## 2. Material and Methods

### 2.1. Bacteria and Cell Lines

The clinical isolate* P. stuartii* NK was obtained from the strain collection of Kazan Federal University, Russia. 16S ribosomal RNA (rRNA) gene sequencing and mass spectrometry on the MALDI BioTyper (Bruker Daltonik) were performed for bacterial strain identification. Luria-Bertani (LB) broth containing (per L) 10 g tryptone, 5 g yeast extract, and 5 g NaCl has been used to maintain the bacterial cells. Bacteria were grown at 37°C with aeration (shaker Braun, Germany).

HeLa-M cells were used as a mammalian cell model for the experiment and were cultivated in Dulbecco's Modified Eagle Medium (DMEM; Gibco®, Grand Island, NY, USA) supplemented with 10% fetal bovine serum (FBS; Gibco, Grand Island, NY, USA) and 20 mM l-glutamine at 37°C under 5% CO_2_ until subconfluent density.

All experiments were performed in duplicate.

### 2.2. Swarming Motility Assay

Swarming was determined as described by Li et al. [14]. A single colony was isolated and grown overnight at 37°C in LB. Approximately 10^8^ colony forming units (CFU) was inoculated at the center of Petri plates containing LB plus 0.5% of agar. The plates were incubated for up to 24 h at 37°C.

### 2.3. Bacterial Growth Assays

LB culture medium has been used for the cultivation of* Providencia stuartii* NK. Subcultures were obtained and incubated in a shaker up to 72 h at 37°C. The 4 h (early log phase), 12 h (late log phase), 24 h (stationary phase), 48 h, and 72 h (death phase) subcultures were harvested by centrifugation, resuspended in PBS, and measured until the optical density (OD) 620 nm reached 0.2 or 0.5 in order to calculate the needed number of bacteria for each experiment. OD620 of 0.2 = 2.5 × 10^7^ CFU/ml; OD620 of 0.5 = 1.4 ± 0.1 × 10^8^ CFU/ml. An aliquot of the diluted subculture was added to each tissue culture plate well.

### 2.4. Bacterial Adhesion* In Vitro* Assay

Adhesion assay was performed essentially as described [15]. Briefly, subconfluent HeLa-M cell monolayers in 12-well cell culture plates were infected and incubated with* P. stuartii* NK suspension at a cell: bacteria ratio of 1 : 50 at 37°C for 2 h. After this incubation, cells were washed three times with phosphate-buffered saline (PBS), pH 7.4 (Sigma, USA). To lyse the eukaryotic cells and detach the adherent bacteria, 100 *μ*l of 1% Triton X-100 was added and incubated for 10 minutes at room temperature. 900 *μ*l of LB medium was then added. Subsequently, serially diluted suspensions were plated on LB-agar plates, and bacteria adhering to the HeLa-M cells were quantified.

### 2.5. Gentamicin Protection (Invasion) Assay

Invasion assay was performed essentially as described [16]. Briefly, host cells were seeded into 6-well plates and grown to subconfluence. Just before infection, the cell culture medium in each well was replaced with 1000 *μ*l of fresh, prewarmed medium. In three sets of triplicate wells, HeLa-M cells were infected with a MOI of 10–100 bacteria per host cell. Bacterial contact with host cells was expedited by centrifugation of plates at 2000*g* for 5 min. After 2 h of incubation at 37°C, cells were washed and detached from wells by treating with 0.25% trypsin-EDTA (Sigma). The resulting suspension was incubated for another 2 h in medium containing 100 *μ*g/ml membrane-impermeable bactericidal antibiotic gentamicin (Sigma) to kill any extracellular bacteria. Cells were then lysed in 4.5% deoxycholate and plated on LB-agar plates. Invasion frequencies were calculated as the number of bacteria surviving incubation with gentamicin divided by the total number of bacteria present just before addition of gentamicin.

### 2.6. Scanning Electron Microscopy

For SEM, HeLa-M cells were grown on glass coverslips and infected with bacteria for 30–120 min at 37°C and subsequently were washed and fixed in 2% glutaraldehyde in PBS buffer (Sigma, USA). Samples were then dehydrated in ascending concentrations of ethyl alcohol, coated with Au/Pd alloy using Quorum Q150T ES vacuum coater, and examined using a scanning electron microscope Merlin (Carl Zeiss, Germany). The microscopy was performed in the Interdisciplinary Center for Analytical Microscopy, KFU.

### 2.7. Confocal Microscopy

To visualize the invasion, confocal fluorescence microscopy was used. HeLa-M cells were grown for 2 days on coverslips and incubated with bacteria according to the procedure described above. Cells were washed with PBS 3 times to remove noninvaded bacteria and fixed in a 3.7% formalin solution for 10 minutes. Further, the preparations were washed from formalin and treated in 0.1% Triton X-100 (Maersk) solution for 5 minutes and again washed with PBS 3 times. Next, the preparations were stained with rhodamine-phalloidin to visualize actin filaments for 15 minutes in the dark and again stained with a solution of DAPI (Sigma), which stains the DNA of bacterial and eukaryotic cells.

The preparations were enclosed in a solution preventing the burning of the fluorescent dye (mounting medium, Sigma) and analyzed by a Leica TCS SL laser scanning microscope (Carl Zeiss, Germany). Blue fluorescence (DAPI) was excited with a laser at a wavelength of 405 nm and red (rhodamine-phalloidin) with He-Ne laser with a wavelength of 543 nm. The DAPI and rhodamine-phalloidin fluorescence was scanned separately using the Leica Confocal Software. Image analysis was performed using the program “WCIF Image J 1.37I” (National Institute of Health, Maryland, USA).

### 2.8. Statistical Analysis

Statistical processing of the results was performed using Microsoft Office Excel standard package by calculating the standard deviation (*σ*). The results were considered reliable for rms deviation *σ* < 10%. As a criterion for the reliability of the obtained differences, Student's test was used, taking *P* < 0.05 for an authoritative level of value.

## 3. Results and Discussion

### 3.1. Identification of Providencia Clinical Isolate

*P. stuartii* are gram-negative, motile bacteria of the Enterobacteriaceae family. They are recognized as opportunistic pathogens in humans and mainly cause urinary tract infections, particularly in patients with long-term indwelling urinary catheters or extensive severe burns [17]. However, the data on the pathogenic potential f* P. stuartii* with the host organism are not full and still being collected. To assess the pathogenicity and evaluate the potential virulence factors of* P. stuartii* for humans, the clinical isolate has been chosen.

Identification of* Providencia* clinical isolate was done by MALDI BioTyper and 16S rRNA gene sequencing. The closest strain as determined by mass spectroscopy analysis is* P. stuartii* 110912_21 LLH (score value 2.5). BLAST analysis of 16S rRNA gene sequencing detected 99% similarity with* P. stuartii* MRSN 2154. Thus, both methods confirmed that this strain belongs to* P. stuartii* and was herein named NK.

### 3.2. Scanning Electron Microscopy of Swarming* P. stuartii* NK Cells

It is known that bacteria have evolved an abundance of mechanisms to engage with host cells and manipulate their cellular signaling programs to facilitate colonization [18]. As a consequence of surface sensing physiological changes may trigger the recruitment of planktonic bacteria and favor interbacterial interactions between the surface-attached and the recruited bacterium. The outcome of such indirect surface associations can be the emergence of bacterial colony [19].

Flagella-mediated motility is relevant to the process of bacterial colonization which is a complex phenotype essential for establishing disease. Within the various types of bacterial movement, swarming motility is used by bacterial cells to move along solid substrates and is of great importance in the colonization process [20].

**Figure 1:**
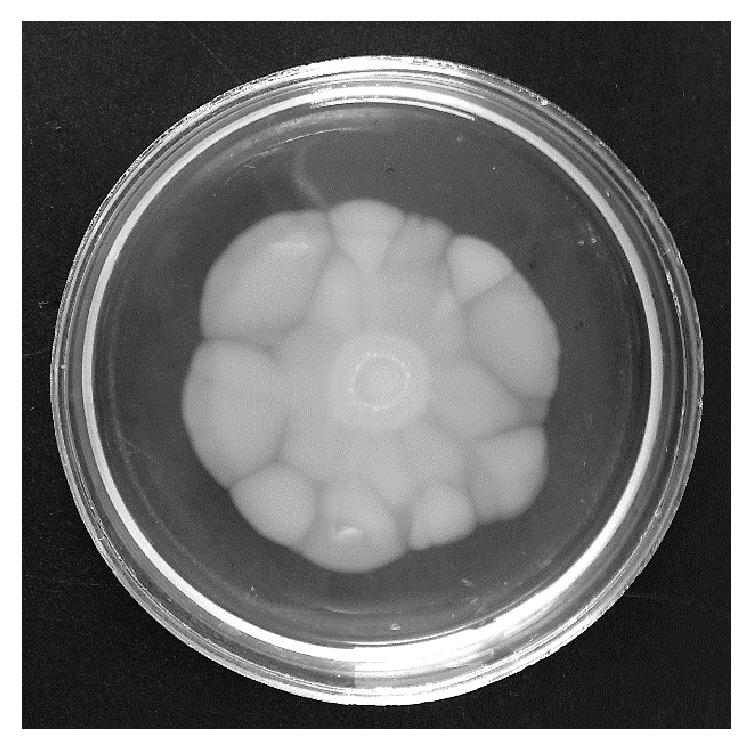
Swarming motility of* P. stuartii* NK on LBA medium containing 0.5% of agar. The cultivation temperature: 37°C. Time of cultivation: 24 hours.

It was shown that* P. stuartii* NK strain is not capable of swarming over the surface of very dense substrates; therefore, bacterial motility was studied on LBA medium with an agar content of 0.5% (Figure 1).

Well known swarming bacteria* Proteus mirabilis* are able to swarm on the surface of solid media (1.8–2% agar) [21]. At the same time, many other types of bacteria are not capable of swarming on such “hard” environments. As can be seen from Figure 1, the colony of the swarming* P. stuartii* NK has irregular edges and does not resemble the colony of* P. mirabilis*.

**Figure 2:**
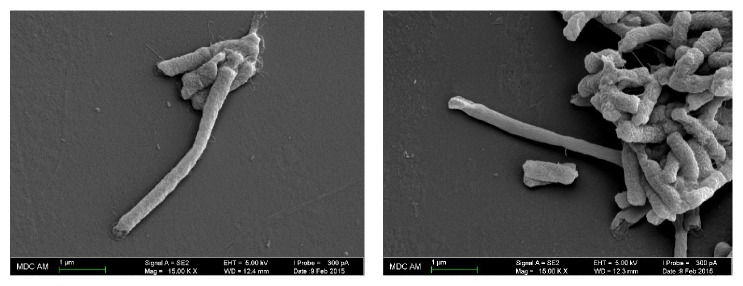
SEM-images of* P. stuartii* NK cells from the colony on 0.5% agar illustrating morphological changes of bacteria during swarming motility. Magnification 15. 00 K X.

It is known that* P. mirabilis* forms colonies with concentric rings, which reflects the periodic nature of swarming bacterial cells. In addition, many articles demonstrate that* P. mirabilis* culture undergoes cell differentiation [22]. The length of* P. mirabilis* swarming cells may exceed the length of vegetative cells 20–40 times [23]. At the same time, other bacteria, such as* S. marcescens*, undergo a much less pronounced change of cell morphology [24]. To investigate the ability of* P. stuartii* NK to differentiate during swarming process, SEM of culture cells from different parts of the swarming colony was performed (Figure 2).

Thus, it was demonstrated that* P. stuartii* NK cells undergo morphological changes on semisolid media and 7 times longer cells appear. However, the number of elongated cells is not numerous.

### 3.3. Adhesion onto HeLa-M Epithelial Cell Line by* P. stuartii* NK

A key stage in the interaction of a pathogen with a susceptible organism is the “attachment” of the pathogen to the surfaces of the host [25]. A necessary state in the ultimate production of disease by microbial pathogens is the ability to adhere to host surfaces such as mucous membranes and gastric and intestinal epithelial or endothelial tissue [26]. Therefore, it is a common trait of microbial pathogens to express adherence factors responsible for recognizing and binding to specific receptor moieties of cells, thus enabling the bacteria to resist host strategies that would impede colonization. Specific adhesion to tissue cells is therefore considered an essential virulence factor for most bacterial pathogens [27].

**Figure 3:**
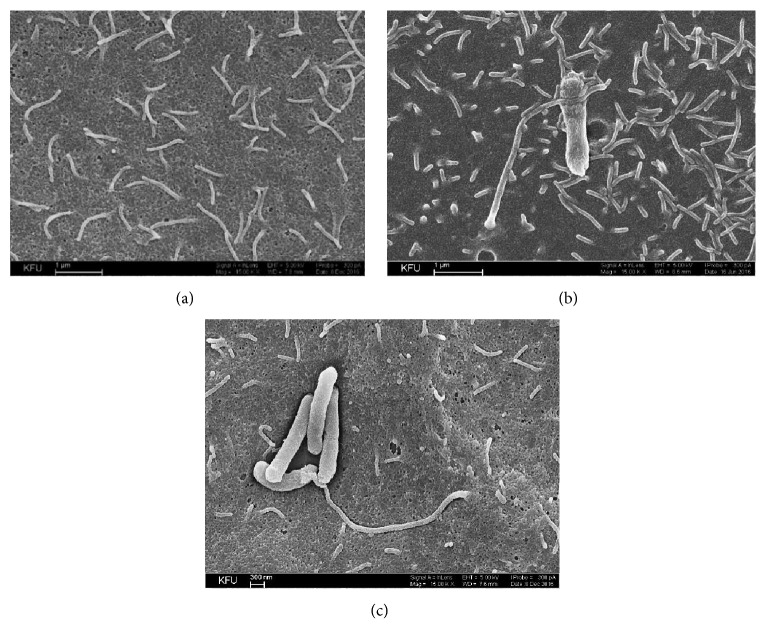
SEM-images of* P. stuartii* NK adhesion onto HeLa-M cells. Subconfluent HeLa-M monolayers were infected with* P. stuartii* NK at a ratio of 50 bacterial cells to 1 eukaryotic cell at 37°C. (a) Control (HeLa-M cells were not infected with bacteria); (b) 30 minutes of postinfection with* P. stuartii* NK; (c) 60 minutes of postinfection with* P. stuartii* NK. Magnification 15. 00 K X.

Scanning electron microscopy (SEM) has been successfully used to image microscopic living organisms because of its high resolution and magnification [28]. It shows in detail the surface morphology which may allow analyzing cell to cell interactions. Since bacterial adhesion onto epithelial cells can be a preliminary step in colonization or invasion, we have examined the ability of* P. stuartii* NK strain to adhere on the surface of HeLa-M epithelial cells by SEM. SEM of culture cells fixed after infection with* P. stuartii* NK revealed that the host cells form contacts with bacteria after 30 minutes of postinfection (Figure 3).

It can be assumed that the dense contact involving microvilli is important for further invasion of bacteria. Kwok et al. (2002) found that* H. pylori* invasion of AGS cells involves close contact with microvilli on the cell membrane. Moreover, the first mode of entry is illustrated by* Listeria* and* Yersinia*, and it involves the formation of cell protrusions in tight contact with the bacterial surface [29]. As can be seen from Figure 3, to form a contact with the bacterium microvilli become elongated. Such findings are similar to the effects observed previously by other researches. For instance, in contact with* Helicobacter pylori*, some epithelial microvilli seemed to have extended beyond their normal length. It was not clear, however, whether this effect was a consequence or a prerequisite of the bacterial contact [30]. At a longer incubation, a cytotoxic effect could be also observed, which is manifested itself in a cellular damage around the bacteria.

Bacterial adhesion depends on various factors: surface properties, culture media, temperature, exposure time, age, and bacterial concentration [31]. To determine the effective conditions for the adhesion of* P. stuartii* NK onto epithelial cells HeLa-M cell culture has been used as well. Our results on quantitative adhesion assay indicate that the adhesion rate depends on the age of the culture.

**Figure 4:**
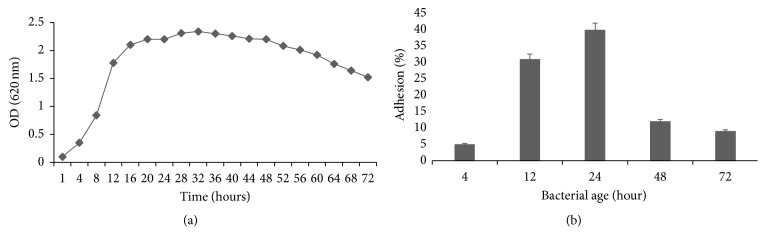
(a) Growth profile for* P. stuartii* NK (37°C, LB). (b) Adherence of bacteria* P. stuartii* NK to HeLa-M cells, depending on the age of the bacterial culture. Subconfluent HeLa-M monolayers were infected with* P. stuartii* NK at a ratio of 50 bacterial cells to 1 eukaryotic cell for 2 hours at 37°C.

The* P. stuartii* NK adherence (Figure 4) was the highest during the stationary phase (24 hours). In the stationary phase, the total number of viable microorganisms remains constant. This may result from a balance between cell division and cell death, or the population may simply cease to divide but remain metabolically active [32].

The growth phase of cells has also been a source of variations in the literature. Some studies have described the adhesive nature of cells at mid-exponential phase [33] and stationary growth phase [34], whereas others have not attempted to characterize the exact time of growth phase, preferring to define the time of growth as “overnight.”

According to our findings, the percentage of* P. stuartii* NK bacterial adhesion onto HeLa-M cells reached 40% during stationary phase. In the case of uropathogenic* Escherichia coli* (UPEC) the percentage of adhesion onto bladder epithelial cells is just below 50%. It was established that the type 1 pilus (FimH) mediates the adhesion of UPEC and initiates bacterial internalization or invasion [35].

### 3.4. Invasion into HeLa-M Epithelial Cell Line by* P. stuartii* NK

**Figure 5:**
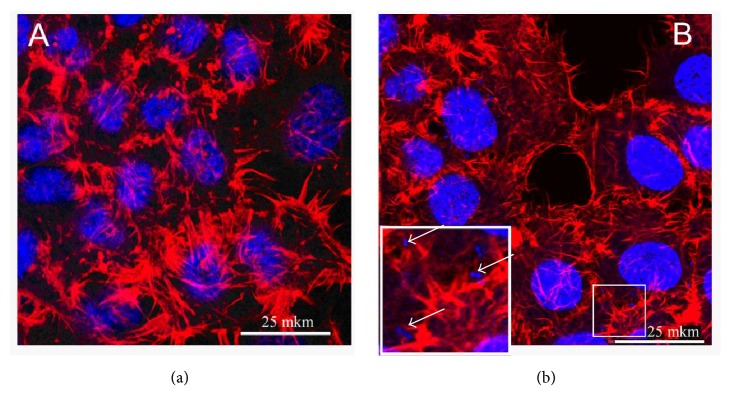
Confocal microscopy of* P. stuartii* NK invasion into HeLa-M cells by confocal microscopy. Subconfluent HeLa-M monolayers were infected with* P. stuartii* NK at a ratio of 50 bacterial cells to 1 eukaryotic cell for 2 hours at 37°C. (a) Control (HeLa-M cells were not infected with bacteria); (b) Experiment (HeLa-M cells were infected with bacteria for 120 minutes). Actin cytoskeleton was stained with rhodamine-phalloidin (red); DNA of cell nuclei and bacteria were stained with DAPI (blue). Inset shows intracellular bacteria (white arrows).

The internalization of bacterial pathogens into nonphagocytic cells has been increasingly investigated and recognized as playing an essential role in bacteria—host-cell interactions [36]. An intracellular lifestyle provides diverse advantages for bacterial pathogens: they become inaccessible to humoral and complement-mediated attack; they avoid shear stress-induced clearance and get access to a wide range of nutrients which provides additional metabolic pathways to use [37]. To analyze the ability of* P. stuartii* NK to invade the host cells, confocal microscopy has been used (Figure 5).

Figure 5 shows that bacterial cells localized in the cytoplasm are seen inside the HeLa-M cells. Also, some changes in the cytoskeleton organization of eukaryotic cells can be seen. Such findings correlate with the knowledge that in order to induce their own uptake intracellular bacteria interact with cytoskeleton [38].

**Figure 6:**
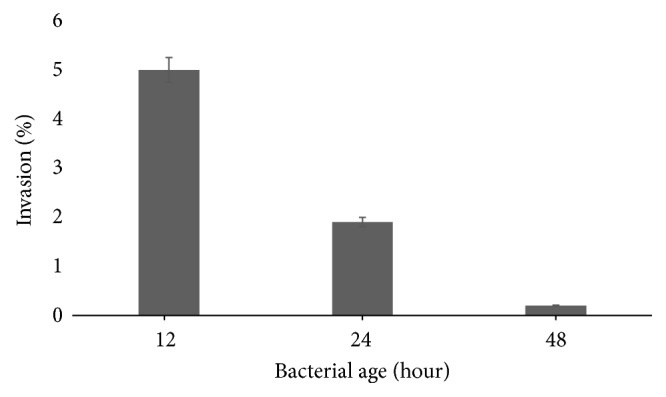
Efficiency of* P. stuartii* NK invasion into HeLa-M cells depending on the stage of bacterial culture growth. Subconfluent HeLa-M monolayers were infected with* P. stuartii* NK at a ratio of 100 bacterial cells to 1 eukaryotic cell for 2 hours at 37°C.

To confirm that bacterial internalization of* P. stuartii* NK is correlated with the age of bacterial culture as well as adhesion, gentamicin protection assay was performed at 2 hours after infection of the HeLa-M epithelial monolayer. The results showed that the bacteria had the highest invasive potential on the late log growth phase (Figure 6), 5% of the invaded cells. In the log phase, cells in the culture are synthesizing new components [39], and apparently this phase plays a certain role in the bacterial invasion into eukaryotic cells. When 24- and 48-hour cultures were used, the proportion of invaded cells did not exceed 2% of the initial infectious dose.

**Figure 7:**
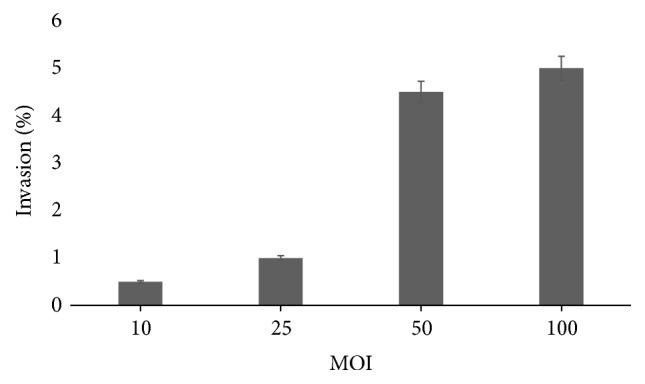
Efficiency of* P. stuartii* NK invasion into HeLa-M cells depending on the multiplicity of infection, 12-hour culture. Subconfluent HeLa-M monolayers were infected with* P. stuartii* NK at the different ratios of bacterial to eukaryotic cells for 2 hours at 37°C.

Usually, bacterial invasion is affected by an infective dose or MOI: the number of bacterial cells per 1 eukaryotic cell [40]. For bacteria* Campylobacter upsaliensis* it has been demonstrated that the efficiency of invasion was highest at an MOI of 10 and then decreased slightly as the MOI increased to 1000 [41]. In the case of* Acinetobacter baumannii*, the MOI of 100 was optimal in the cell invasion assay [42]. In order to determine the contribution of the infective dose to the invasion process of* P. stuartii* NK, the different ratios of epithelial to bacterial cells were incubated at 37°C for 2 hours. This experiment showed that bacteria* P. stuartii* NK invade effectively HeLa-M cells at the ratios of eukaryotic to bacterial cells 1 : 50 and 1 : 100 (Figure 7).

In the highest infective dose at the ratio of eukaryotic to bacterial cells 1 : 100, percentage of invasion reached 5% only, which means that bacteria* P. stuartii* NK require a high infective dose for successful internalization into epithelial monolayer. The obtained data indicate a low invasive potential of the investigated bacteria, which is typical for opportunistic pathogens. Also, the cytotoxic effect of* P. stuartii* NK has not been detected, which makes it possible to use this strain as an object for researching the molecular mechanisms of adhesion and invasion within opportunistic bacteria of* Enterobacteriaceae* family.

Apparently, longer incubation time may also stimulate the higher proportion of internalized bacteria. Eugène et al. in the experiments with* Neisseria meningitidis* varied the time of incubation with endothelial monolayers up to 8 hours [43]. But in some experiments such elongations of the incubation time reduced the number of invaded bacteria because a cytotoxic effect and cell damage were observed [44].

This investigation may increase our insight into the virulence factors used by opportunistic bacteria. Studies on the pathogenic potential of* P. stuartii* will be continued, because some essential questions remain unanswered.

## 4. Conclusions

The results on our studies showed that opportunistic bacteria* P. stuartii* are able to migrate over viscous surfaces and adhere to and invade HeLa-M cell culture effectively. These properties directly depend on the stage of the bacterial growth. The stationary growth phase is optimal for adhesion, while the late log phase is optimal for invasion. In addition, an adequate infection dose of* P. stuartii* NK is critical to invade the eukaryotic cells effectively.

## References

[1] Wie S.-H. (2015). Clinical significance of providencia bacteremia or bacteriuria. *Korean Journal of Internal Medicine*.

[2] De Vecchi E., Sitia S., Romanò C. L., Ricci C., Mattina R., Drago L. (2013). Aetiology and antibiotic resistance patterns of urinary tract infections in the elderly: a 6-month study. *Journal of Medical Microbiology*.

[3] Aubert D., Naas T., Lartigue M.-F., Nordmann P. (2005). Novel genetic structure associated with an extended-spectrum *β*-lactamase blaVEB gene in a Providencia stuartii clinical isolate from Algeria. *Antimicrobial Agents and Chemotherapy*.

[4] Tumbarello M., Citton R., Spanu T. (2004). ESBL-producing multidrug-resistant Providencia stuartii infections in a university hospital. *Journal of Antimicrobial Chemotherapy*.

[5] Krake P. R., Tandon N. (2004). Infective endocarditis due to Providenda stuartii. *Southern Medical Journal*.

[6] Simon C., Dieli M., Brucato A. (2010). Bacterial pericarditis due to providencia stuartii: An atypical case of relapsing pericarditis. *Circulation*.

[7] Unverdi S., Akay H., Ceri M. (2011). Peritonitis due to Providencia stuartii. *Peritoneal Dialysis International*.

[8] Sipahi O. R., Bardak-Ozcem S., Ozgiray E. (2010). Meningitis due to Providencia stuartii. *Journal of Clinical Microbiology*.

[9] Pizarro-Cerdá J., Cossart P. (2006). Bacterial adhesion and entry into host cells. *Cell*.

[10] McClure E. E., Chávez A. S. O., Shaw D. K. (2017). Engineering of obligate intracellular bacteria: Progress, challenges and paradigms. *Nature Reviews Microbiology*.

[11] Johnson D. E., Lockatell C. V., Hall-Craigs M., Mobley H. L. T., Warren J. W. (1987). Uropathogenicity in rats and mice of Providencia stuartii from long-term catheterized patients. *The Journal of Urology*.

[12] Khashe S., Scales D. J., Abbott S. L., Janda J. M. (2001). Non-invasive Providencia alcalifaciens strains fail to attach to HEp-2 cells. *Current Microbiology*.

[13] Mobley H. L. T., Chippendale G. R., Tenney J. H., Warren J. W. (1986). Adherence to uroepithelial cells of Providencia stuartii isolated from the catheterized urinary tract. *Journal of General Microbiology*.

[14] Li C., Louise C. J., Shi W., Adler J. (1993). Adverse conditions which cause lack of flagella in Escherichia coli. *Journal of Bacteriology*.

[15] Letourneau J., Levesque C., Berthiaume F., Jacques M., Mourez M. (2011). In vitro assay of bacterial adhesion onto mammalian epithelial cells. *Journal of Visualized Experiments*.

[16] Elsinghorst E. A. (1994). Measurement of invasion by gentamicin resistance. *Methods in Enzymology*.

[17] Armbruster C. E., Smith S. N., Yep A., Mobley H. L. T. (2014). Increased incidence of urolithiasis and bacteremia during proteus mirabilis and providencia stuartii coinfection due to synergistic induction of urease activity. *The Journal of Infectious Diseases*.

[18] Boyle E. C., finlay B. B. (2003). Bacterial pathogenesis: Exploiting cellular adherence. *Current Opinion in Cell Biology*.

[19] Stones D. H., Krachler A. M. (2016). Against the tide: The role of bacterial Adhesion in host colonization. *Biochemical Society Transactions*.

[20] Fraser G. M., Hughes C. (1999). Swarming motility. *Current Opinion in Microbiology*.

[21] Rather P. N. (2005). Swarmer cell differentiation in Proteus mirabilis. *Environmental Microbiology*.

[22] Verstraeten N., Braeken K., Debkumari B. (2008). Living on a surface: swarming and biofilm formation. *Trends in Microbiology*.

[23] Morgenstein R. M., Clemmer K. M., Rather P. N. (2010). Loss of the waaL O-antigen ligase prevents surface activation of the flagellar gene cascade in Proteus mirabilis. *Journal of Bacteriology*.

[24] Alberti L., Harshey R. M. (1990). Differentiation of Serratia marcescens 274 into swimmer and swarmer cells. *Journal of Bacteriology*.

[25] Nielubowicz G. R., Mobley H. L. T. (2010). Host-pathogen interactions in urinary tract infection. *Nature Reviews Urology*.

[26] Finlay B. B., Falkow S. (1997). Common themes in microbial pathogenicity revisited. *Microbiology and Molecular Biology Reviews*.

[27] Klemm P., Schembri M. A. (2000). Bacterial adhesins: function and structure. *International Journal of Medical Microbiology*.

[28] Asahi Y., Miura J., Tsuda T. (2015). Simple observation of Streptococcus mutans biofilm by scanning electron microscopy using ionic liquids. *AMB Express*.

[29] Kwok T., Backert S., Schwarz H., Berger J., Meyer T. (2002). Specific entry of Helicobacter pylori into cultured gastric epithelial cells via a zipper-like mechanism. *Infection and Immunity*.

[30] Galan J. E., Zhou D. (2000). Striking a balance: Modulation of the actin cytoskeleton by Salmonella. *Proceedings of the National Acadamy of Sciences of the United States of America*.

[31] Garrett T. R., Bhakoo M., Zhang Z. (2008). Bacterial adhesion and biofilms on surfaces. *Progress in Natural Science*.

[32] Pletnev P., Osterman I., Sergiev P., Bogdanov A., Dontsova O. (2015). Survival guide: Escherichia coli in the stationary phase. *Acta Naturae*.

[33] Walker S. L., Redman J. A., Elimelech M. (2004). Role of cell surface lipopolysaccharides in escherichia coli K12 adhesion and transport. *Langmuir*.

[34] Bruinsma G. M., Rustema-Abbing M., Van Der Mei H. C., Busscher H. J. (2001). Effects of cell surface damage on surface properties and adhesion of Pseudomonas aeruginosa. *Journal of Microbiological Methods*.

[35] Martinez J., Mulvey M., Schilling J., Pinkner J., Hultgren S. (2000). Type 1 pilus-mediated bacterial invasion of bladder epithelial cells. *The European Molecular Biology Organization Journal*.

[36] Konkel M. E., Samuelson D. R., Eucker T. P., Shelden E. A., O'Loughlin J. L. (2013). Invasion of epithelial cells by Campylobacter jejuni is independent of caveolae. *Cell Communication and Signaling*.

[37] Ribet D., Cossart P. (2015). How bacterial pathogens colonize their hosts and invade deeper tissues. *Microbes and Infection*.

[38] Bonazzi M., Lecuit M., Cossart P. (2009). Listeria monocytogenes internalin and E-cadherin: From structure to pathogenesis. *Cellular Microbiology*.

[39] Walker S. L., Hill J. E., Redman J. A., Elimelech M. (2005). Influence of growth phase on adhesion kinetics of Escherichia coli D21g. *Applied and Environmental Microbiology*.

[40] Edwards A. M., Massey R. C. (2011). Invasion of human cells by a bacterial pathogen. *Journal of Visualized Experiments*.

[41] Mooney A., Byrne C., Clyne M., Johnson-Henry K., Sherman P., Bourke B. (2003). Invasion of human epithelial cells by Campylobacter upsaliensis. *Cellular Microbiology*.

[42] Choi C. H., Lee J. S., Lee Y. C., Park T. I., Lee J. C. (2008). Acinetobacter baumannii invades epithelial cells and outer membrane protein A mediates interactions with epithelial cells. *BMC Microbiology*.

[43] Eugène E., Hoffmann I., Pujol C., Couraud P.-O., Bourdoulous S., Nassif X. (2002). Microvilli-like structures are associated with the internalization of virulent capsulated Neisseria meningitidis into vascular endothelial cells. *Journal of Cell Science*.

[44] Cano V., Moranta D., Llobet-Brossa E., Bengoechea J. A., Garmendia J. (2009). Klebsiella pneumoniae triggers a cytotoxic effect on airway epithelial cells. *BMC Microbiology*.

